# Severity of specific language impairment predicts delayed development in number skills

**DOI:** 10.3389/fpsyg.2013.00581

**Published:** 2013-09-03

**Authors:** Kevin Durkin, Pearl L. H. Mok, Gina Conti-Ramsden

**Affiliations:** ^1^School of Psychological Sciences and Health, University of StrathclydeGlasgow, UK; ^2^School of Psychological Sciences, Communication and Deafness, The University of ManchesterManchester, UK

**Keywords:** number skills, number development, specific language impairment (SLI), cognitive factors, linguistic abilities

## Abstract

The extent to which mathematical development is dependent upon language is controversial. This longitudinal study investigates the role of language ability in children's development of number skills. Participants were 229 children with specific language impairment (SLI) who were assessed initially at age 7 and again 1 year later. All participants completed measures of psycholinguistic development (expressive and receptive), performance IQ, and the Basic Number Skills subtest of the British Ability Scales. Number skills data for this sample were compared with normative population data. Consistent with predictions that language impairment would impact on numerical development, average standard scores were more than 1 *SD* below the population mean at both ages. Although the children showed improvements in raw scores at the second wave of the study, the discrepancy between their scores and the population data nonetheless increased over time. Regression analyses showed that, after controlling for the effect of PIQ, language skills explained an additional 19 and 17% of the variance in number skills for ages 7 and 8, respectively. Furthermore, logistic regression analyses revealed that less improvement in the child's language ability over the course of the year was associated with a greater odds of a drop in performance in basic number skills from 7 to 8 years. The results are discussed in relation to the interaction of linguistic and cognitive factors in numerical development and the implications for mathematical education.

## Introduction

Developing competence with number is fundamental to everyday life and to education. One of the best predictors of subsequent academic attainment is children's level of rudimentary number skills at school entry (Duncan et al., 2007). Understanding how children deal with this domain and what factors are associated with their learning are topics of theoretical and practical significance. Although most scientists in this area agree that developing number skills depends on complex neural, cognitive, linguistic and interpersonal abilities, the relative contributions of different capacities, and their interrelationships, are controversial and difficult to disentangle. Many researchers have argued that linguistic or linguistically-supported processes, such as labeling, articulating and scaffolding, are critical to mastering basic number, and that conceptual knowledge of the domain is organized, memorized, shared and built upon via language (Durkin, 1993; Wiese, 2003; Carey, 2004; Musolino, 2004; Ginsburg, 2009; Mix, 2009; Negen and Sarnecka, 2012). In contrast, others argue that numerical cognition has phylogenetic and ontogenetic origins independent of the language capacity (Gelman and Butterworth, 2005; Nieder and Dehaene, 2009).

Children with specific language impairment (SLI) are of particular interest in this context (Fazio, 1996, 1999; Arvedson, 2002; Cowan et al., 2005; Donlan et al., 2007; Kleemans et al., 2011, 2012; Nys et al., 2013). Individuals with SLI fall within the normal range of cognitive abilities, show no evidence of neurological damage or hearing impairment yet, relative to peer norms, have deficits in either or both of expression and comprehension of language (Bishop, 1997; Conti-Ramsden and Durkin, 2011). SLI is a common condition affecting some 7% of 5-year-old children (Tomblin et al., 1997). Children with this condition provide a naturally-occurring test of to what extent number skills development is possible when language abilities are compromised but cognitive abilities are within the typical range. In this report, we examine the longitudinal relationship between language ability and number skills development in children with SLI. To explain the background to the study, first we summarize evidence on the relationship between language and number development in the preschool and early school years, and then we describe previous research on number development in children with SLI.

Some important number-related skills emerge before language. Preverbal human infants can discriminate among numbers (Strauss and Curtis, 1981; Wynn, 1992; Cordes and Brannon, 2009), though the basis on which they do so is controversial (Ansari and Karmiloff-Smith, 2002; Sophian, 2007; Izard et al., 2009; Núñez, 2011). While early perceptual abilities may provide a foundation on which to map some number terms, subsequent developments are—for typically developing (TD) children—inevitably interwoven with linguistic experiences and language development (Spelke and Tsivkin, 2001). Number words are common in everyday parental input to infants and toddlers (Durkin et al., 1986; Fuson, 1988; Bloom and Wynn, 1997; Tare et al., 2008). Furthermore, variation in the extent and quality of number talk in parental speech has been found to predict developments among pre-schoolers in conceptual number knowledge, such as cardinal meanings (Linnell and Fluck, 2001; Levine et al., 2010; Mix et al., 2012).

Developments in language skills are clearly associated with developments in numerical and mathematical abilities. Negen and Sarnecka (2012) showed that the size of a child's general nominal vocabulary (both expressive and receptive) is positively associated with number-concept acquisition, suggesting that noun learning could assist children in discovering the meanings of number words. For example, to understand the number word in a caregiver's utterance such as “Look! There's a mommy duck with three baby ducks!” (Negen and Sarnecka, 2012, p. 2020), it helps if the child already knows what the noun *duck* means and how one creates plural forms. O'Neill et al. (2004) found that measures of narrative competence at 3- to 4- years were predictive of mathematics ability assessed 2 years later.

As children begin to use number words with increasing accuracy in everyday settings, advances follow in their abilities to apply number concepts in contexts where they are trained or tested on number understanding (Mix et al., 2005; Palmer and Baroody, 2011; Reikerås et al., 2012). When children participate in educational contexts, number and early mathematical concepts receive more, and increasingly deliberate, attention (Ginsburg, 2009). Much of this is mediated through language: the verbal instructions of the teacher, the vocabulary and syntax of texts and other school materials (Durkin and Shire, 1987, 1991; Ellerton and Clarkson, 1996; Ginsburg, 2009). Cross-linguistic investigations of number skills development show that children's performances can be helped or hindered by particular features of the way a given language encodes number (Seron and Fayol, 1994; Roberts and Gathercole, 2006; Salehuddin and Winskel, 2009; Zuber et al., 2009; Helmreich et al., 2011; Pixner et al., 2011).

With development, it is increasingly difficult to determine which variables affect progress in number and mathematical abilities, and in practice most children using their number skills will require both computational and linguistic application. How, then, do children whose linguistic abilities are impaired handle this domain?

Children who have difficulties in using language may face particular challenges in advancing their numerical understanding. Indeed, children with SLI face challenges in many areas of development and education (Bishop and Adams, 1990; Conti-Ramsden et al., 2009; Durkin et al., 2009; St. Clair et al., 2010). Difficulties in decoding others' language, in formulating and producing utterances, and in processing textual materials pose considerable burdens when dealing with novel concepts and problem-solving procedures. As Gelman and Butterworth (2005) comment, it would be surprising if there were no effects of language on numerical cognition, and even proponents of independent origins of numerical ability acknowledge that language facilitates the use of numerical concepts.

Previous research with children with SLI confirms that they do lag behind typical peers in progress in number and mathematical abilities, though this depends to some extent on which abilities are tested. Fazio (1994, 1996, 1999) followed a small group of children with SLI from preschool to age 9 years. She found that the children were late in acquiring counting skills and, although they did gradually develop these, they continued to experience difficulties with related rote memory tasks (such as multiplication tables), which she interpreted as indicating that storage or retrieval problems underlie the children's difficulties in this domain. Nelson et al. (2011) found that 4-year-olds with severe language delay scored 1 *SD* below norms on a maths test measuring counting skills and simple addition and subtraction. Kleemans et al. (2011, 2012) showed that naming speed (the ability to retrieve linguistic information from long-term memory) was associated with early verbal numeracy skills in children with SLI (but not in TD children). On the other hand, naming speed did not predict performance on numerical estimation tasks (identifying the location of a number on a number line), which are less verbal. Arvedson (2002) found that on some number tasks designed to minimize verbal processing, 3- to 5-year-old children with SLI performed as well as age matched TD peers, and better than language-matched younger children. However, on tasks that required more verbal processing, and particularly when children were encouraged to count, the participants with SLI performed less well than their TD peers. Donlan et al. (2007), working with 8-year-olds with SLI, reported further evidence of severe deficits in both counting tasks and calculation tasks, compared to TD children. Participants with SLI did not differ from age-matched TD children, though, on a task of arithmetical principles in which judgments were required of the correctness or otherwise of abstract symbolic expressions, the most complex but arguably least linguistic of the tasks that they administered.

In one of the largest studies of number skills in primary school aged children with SLI (aged 8 years), Cowan et al. (2005) found that these participants performed worse than age-matched TD comparison children on a range of tasks, including counting, knowledge of addition combinations, basic calculation, story problems, transcoding (reading and writing multi-digit numbers), and relative magnitude judgments. That is, the disadvantage appeared to be pervasive across number tasks. Zero order correlations revealed consistently stronger associations between number task performances and language comprehension than those found for number task performance and either of working memory and non-verbal reasoning. In standard multiple regressions, language comprehension was the best predictor of variation on most of the number tasks. However, Cowan et al. included all participants, including those with typical development, in their regression analyses. While this provides a good test of the importance of language ability across a range of children, it does not examine directly the impact of severity of impairment among those diagnosed as having SLI.

Taken together, previous research demonstrates that children with SLI are at a disadvantage when it comes to working with number. This is evident from their early difficulties with counting, through to a range of other number-related tasks in childhood. The findings are consistent with the assumption that language ability bears in some way on number development in the preschool to early school years. However, most of the available evidence is based on comparisons between children with SLI and TD peers at a particular age point. We lack information on the extent to which language impairment predicts progress in number tasks over time. Kleemans et al. (2012) provide valuable longitudinal data to show that grammatical ability at age 6 does contribute toward the prediction of number skills at age 7, but their study did not measure number skills at both age points, leaving the question of what underpins progress in need of further attention.

In this study, we examined a large sample of children with SLI, collecting their raw and standard scores on a standardized number skills test at two age points (7 and 8 years). We measured their psycholinguistic profiles and non-verbal performance IQ (PIQ) at the outset. We investigated changes in number skills over the period tested. Children receive instruction in number at school, and various outcomes are possible. One, which we will describe as the “separate origins” hypothesis, is that, as operating on numbers involves cognitive processes that are independent of language, continuing progress in number performance should be independent of the severity of impairment; this is not to say that children with language impairments will necessarily display exceptionally strong number skills if they have not done so before, but some improvements in raw scores, and some variations in standard scores, should follow from maturational and educational effects and these should not be predictable on the basis of language abilities. An alternative possibility, which we will call the “language contingent” hypothesis, is that the development of number skills in childhood is influenced by the child's linguistic abilities. Thus, on this account, children with SLI should remain at a disadvantage, reflecting a general lagging behind associated with language delay (cf. Conti-Ramsden et al., 2012). Modest improvements in raw scores could be expected, as the children do improve their language skills albeit at a slow pace, and they are exposed to number education; but, as they find it increasingly difficult to keep up with the language demands associated with number work, standard scores should fall relative to peer norms. The present study was designed to test these competing predictions.

## Materials and methods

### Participants

The participants in this study were originally part of a wider study: the Manchester Language Study (Conti-Ramsden et al., 1997; Conti-Ramsden and Botting, 1999). The original cohort of 242 children, which consisted of 186 boys (76.9%) and 56 girls (23.1%), was recruited from 118 language units in England in 1995, and represented a random sample of 50% of all Year 2 children (approximately 7 years of age) attending language units for at least half of the school week. Language units are specialized classes for children who have been identified with primary speech and language difficulties; the units are usually attached to mainstream schools. Children were excluded from the study if they were reported by their teachers as having frank neurological difficulties, a diagnosis of autism, hearing impairment or a general learning disability. No additional criteria of SLI were used in the selection. Assessment of the children at age 7 after recruitment into the study indicated that the majority met the traditional criteria of SLI, i.e., the standard scores for at least one test of language was less than 1 *SD* below the mean whilst PIQ was within 1 *SD* of the mean, or there was a discrepancy of 40 percentiles or more between the language test scores and PIQ (Conti-Ramsden and Botting, 1999).

A total of 232 of these children were followed up a year later at age 8. For the purpose of this study, only those for whom there were data on their basic number skills measured at both ages have been included in the analyses. This resulted in a total of 229 children, 176 (76.9%) of whom were boys. The mean age of the participants was 7:1 years (6:6–7:9) at wave 1 and 8:1 years (7:5–8:9) at wave 2. The psycholinguistic profiles of the participants at ages 7 and 8 are shown in Table 1. The average standard scores for receptive and expressive languages at both ages were all around 1 *SD* below the population mean, whilst average PIQ was above the population mean at both ages. All children had English as a first language. A small number of children, 27 (11.8%), had exposure to languages other than English at home. Household income data were available for 144 of the participants, with 9.7% coming from households with an annual income of less than £5500 (low income), 24.3% from households earning between £5600 and £10,500 (low-medium income), 19.4% from households earning between £10,600 and £15,500 (medium income), 22.9% from households with an annual income of between £15,600 and £21,000 (medium–high income), and 23.6% from families with an income of over £21,000 (high income).

**Table 1:** **Psycholinguistics profiles at ages 7 and 8**.

	**Age 7**			**Age 8**		
	***N***	**Mean**	***SD***	***N***	**Mean**	***SD***
Receptive language raw scores^a^	229	10.2	3.4	229	12.4	3.2
Receptive language standard scores^a^	229	84.0	10.9	229	86.1	12.3
Expressive language raw scores^b^	219	21.8	7.7	221	25.6	8.4
Expressive language standard scores^b^	219	83.7	10.5	221	84.2	11.4
Receptive and expressive language composite^c^	219	32.1	10.2	221	38.0	10.5
PIQ raw scores^d^	221	19.1	4.6	227	21.8	5.4
PIQ standard scores^d^	221	106.0	14.9	214	108.8	15.5

a TROG.

b Bus Story Test.

c Sum of TROG and Bus Story Test raw scores.

d Raven's Coloured Progressive Matrices.

### Measures

#### Basic number skills

Number abilities were assessed by Test B of the Basic Number Skills subtest of the British Ability Scales (BAS) (Elliot, 1983). The test covers both pre-numerical (matching, classifying) and numerical aspects of number skills. Questions are asked covering the following areas: (1) matching and classifying, using qualitative attributes (e.g., matching spades to buckets based on their sizes); (2) matching and classifying by number (e.g., matching 2 ladybirds which have the same number of spots); (3) one to one correspondence (e.g., matching number of boxes in the picture to numerals); (4) comparison of sets (e.g., comparing picture of carts with more, less and same number of boxes); (5) ordinal aspects of number; (6) knowledge of number names and numerals; (7) counting a set of objects; (8) awareness of number patterns; (9) place value, including the ability to count in tens and knowledge of place value notation; (10) basic understanding of the four arithmetical operations.

For all tasks, children are presented with color picture cues and the instructions of the test are given verbally by the assessor, following the wordings given in the manual. Gestures are also used, e.g., by pointing and circling objects in the booklet. For example, for the question on matching and classifying using qualitative attributes, two pictures cues are used, one with two spades (one big and one small) and one with eight buckets (four big and four small). The child is asked to match spades to buckets based on their sizes. Indicating appropriately, the tester says “Here is a big spade and here is a little spade.” Indicating with a circular motion, s/he then says “Here are the buckets that go with them. Can you show me all the buckets that go with the big spade?” For matching and classifying by number, again using two pictures as cues, one with four yellow ladybirds and one with 12 red ladybirds, the child is asked to match a red and a yellow ladybird which have the same number of spots. The instructions of the test include, “Here are 4 yellow ladybirds,” then indicating to the appropriate ladybirds, the tester says “They may have a lot of spots or (indicating appropriately) very few spots or something in between.” Pointing to the yellow ladybird with nine spots, the tester then asks the child to “Find me the red ladybirds that go with this one.” Similarly, to assess basic understanding of the four arithmetical operations, one of the questions involved showing the child a picture of a box with seven buttons. The tester then says to the child “There used to be 12 buttons in this box. How many have been taken out?”

Testing discontinued after six successive items have been failed. The number of correct answers is summed to give a basic number skills raw score, which can then be converted into a standard score and be assigned an age-relevant percentile.

#### Receptive and expressive language

Receptive language at ages 7 and 8 was assessed using the Test for Reception of Grammar (TROG; Bishop, 1982). This is a test of oral comprehension of syntax in which children are shown four pictures while the examiner reads a sentence. The child is asked to pick the picture that illustrates the sentence. These items begin very simply and progress to more complex grammatical sentences (e.g., “the cat the cow chases is black”). Items are organized into blocks of four grammatically related sentences. The number of blocks passed is noted to give the TROG raw score, which can then be transformed into a standard score. Expressive language at both ages was assessed using the Bus Story Test (Renfrew, 1991), which is part of the Renfrew Language Scales. In this assessment, the examiner tells the child a short story about a bus while the child looks through a book of pictures illustrating the story. The child must then retell the story as accurately as possible using the pictures as cues. Stories are audiotaped, transcribed and scored for the amount of correct information given. Two points are given for information central to the story, and one point for peripheral details, and these are summed to give the “total information score,” which can subsequently be converted into a standard score. The receptive and expressive language raw scores were found to be highly correlated, *r* = 0.62, *p* < 0.001 at age 7, and *r* = 0.61, *p* < 0.001 at age 8. For the purpose of this study, therefore, a composite score representing both receptive and expressive language ability was derived by summing the TROG raw score and the Bus Story “total information score” measured at the corresponding age.

#### Performance IQ

Raven's Coloured Progressive Matrices (Raven, 1986) was used to assess participants' PIQ at both ages. The test is designed for educational and clinical assessment. It can be used satisfactorily with children who have language impairment and is commonly used to assess the PIQ of children with SLI. This test presents the child with a series of patterns from which a “piece” is missing, and the child is asked to choose from six alternative pieces the one which completes the pattern. The test is split into three sets of 12 patterns each and the number of correct answers is summed. This total score is then compared with age-relevant population norms.

### Procedure

Following informed written consent from families, children were visited at school and assessed individually in a quiet room or area with only the participant and a trained researcher present. The battery of psychometric tests was completed as part of the wider study which also included assessments for vocabulary, reading, articulation, and grammatical knowledge. The assessments were conducted in the same order for every child: (1) TROG, (2) BAS Number Skills, (3) BAS Naming Vocabulary (Elliot, 1983), (4) BAS Word Reading (Elliot, 1983), (5) Goldman-Fristoe Test of Articulation (Goldman and Fristoe, 1986), (6) Raven's Coloured Progressive Matrices, (7) Illinois Test of Psycholinguistic Ability: grammatic closure (Kirk et al., 1968), and (8) Renfrew Bus Story Test. In nearly all cases, testing was completed in 1 day at the child's pace and with normal school breaks. Because of the large number of measures used, the numbers of data points available may vary from measure to measure. Both raw scores and standard scores were examined in relation to children's basic number skills. All subsequent analyses used raw scores. All statistical analyses were conducted using Stata/SE 12.0 (StataCorp, 2011). All figures were also plotted using Stata.

## Results

### Basic number skills

Preliminary analyses showed no main effects or interactions involving participant gender or household income; hence, data were pooled across these variables for the main analyses. Descriptive statistics on BAS number skills (raw scores and standard scores) as a function of age are presented in Table 2.

**Table 2:** **Basic number skills at ages 7 and 8**.

	**Age 7**				**Age 8**			
	**Mean**	***SD***	**Min**	**Max**	**Mean**	***SD***	**Min**	**Max**
BAS number skills raw scores	21.0	6.3	2	34	25.8	6.2	0	34
BAS number skills standard scores	82.3	13.0	64	121	80.1	13.4	64	112
BAS number skills percentile^a^	18.6	21.4	<1	92	16.7	19.4	<1	78

N = 229.

a 25 (10.9%) and 48 (21.0%) of the 229 children at ages 7 and 8, respectively scored less than 1 percentile. These cases were coded as having a percentile of 0.5 when the mean percentiles were calculated.

The raw scores rose by an average of 4.8 points between ages 7 and 8, *t*_(228)_ = 14.9, *p* < 0.001, Cohen's *d* = 0.99. The mean standard scores were more than 1 *SD* below the population mean at both ages. The standard scores declined on average 2.2 points, a significant drop, *t*_(228)_ = −3.2, *p* = 0.001, Cohen's *d* = 0.2. Thus, although there had been an improvement in the participants' basic number skills during the year, compared to normative data for TD peers they were on average performing more poorly at age 8 than at age 7. This drop in performance relative to the general population can also be seen in Table 3, which shows the distribution of the standard scores for the two ages. For example, while 16.6% of the children scored 2 *SD* below the population mean at age 7, this rose to 30.1% by age 8.

**Table 3:** **Distribution of basic number skills standard scores at ages 7 and 8**.

**Range of standard scores**	**Age 7 (%)**	**Age 8 (%)**
≤70 (2 *SD* below mean)	16.6	30.1
>70 and ≤77.5 (1.5–2 *SD* below mean)	27.5	18.8
>77.5 and ≤85 (1–1.5 *SD* below mean)	23.1	17.5
>85 and ≤100 (Between 1 *SD* below mean and mean)	23.1	25.3
>100 and <115 (Above mean but below 1 *SD* above mean)	8.3	8.3
≥115 (1 *SD* above mean)	1.3	0
Total	100	100

### Concurrent predictors of basic number skills

Concurrent predictors of performance (using raw scores) at each age were examined first. Using linear regression analysis, covariates were added into the models in two steps: PIQ in the first step, followed by language skills. Given that the distribution of the basic number skills raw scores at age 8 was negatively skewed (skewness = −0.70, kurtosis = 3.31), reflect square root transformations were carried out and the transformed variables were used as the outcome in the linear regression at age 8.

**Figure 1:**
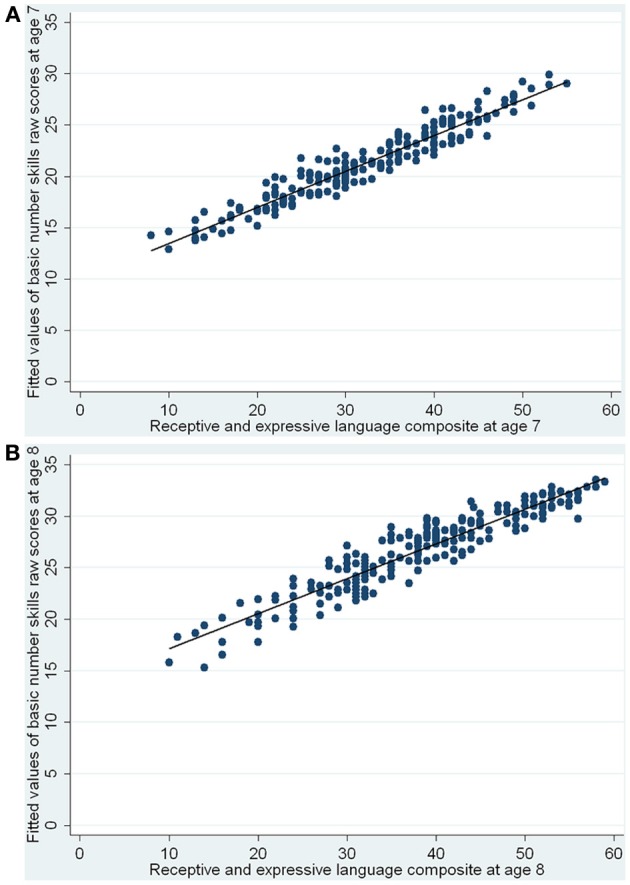
**(A,B)** Fitted values of basic number skills raw scores vs. receptive and expressive language composite—concurrent predictors of basic number skills at ages 7 and 8.

The regression results, shown in Table 4, indicated that PIQ and language scores were significant predictors of concurrent basic number skills at both ages, with better abilities in these areas being associated with better basic number skills. The adjusted *R*^2^ values showed that together they accounted for 36 and 40% of the variance in basic number skills performance at ages 7 and 8, respectively. Comparisons of the standardized regression coefficients also suggested that the effect of language was greater than that of PIQ. After controlling for the effect of PIQ, language skills explained an additional 19 and 17% of the variance in the raw scores for ages 7 and 8, respectively. Figures 1A,B show the fitted (i.e., predicted) values of basic number skills raw scores vs. the language composite, for ages 7 and 8, respectively. At age 8, since the reflect square root transformed number skills raw scores were used as the outcome variable in the linear regression, for easier graphical interpretation, we have re-transformed the predicted values back to the original scale of the raw scores. The figures show that for both ages, the better the language, the higher the predicted values of basic number skills (controlling for PIQ).

**Table 4:** **Linear regression analysis predicting basic number skills performance using concurrent variables at ages 7 and 8**.

	***b***	***SE***	**β**	***R*^2^**	**Adjusted *R*^2^**	**Cohen's *f*^2^**
**RAW SCORES AT AGE 7 (*N* = 212)**						
*Step 1*				0.170	0.166	0.20
PIQ at age 7	0.54^***^	0.08	0.41			
*Step 2*				0.366	0.360	0.58
PIQ at age 7	0.24^**^	0.08	0.18			
Receptive and expressive language composite at age 7	0.30^***^	0.04	0.50			
**TRANSFORMED RAW SCORES AT 8^a^ (*N* = 220)**						
*Step 1*				0.230	0.226	0.30
PIQ at age 8	−0.09^***^	0.01	−0.48			
*Step 2*				0.403	0.398	0.68
PIQ at age 8	−0.05^***^	0.01	−0.25			
Receptive and expressive language composite at age 8	−0.05^***^	0.01	−0.48			

** p < 0.01.

*** p < 0.001.

a Reflect square root transformation of raw scores at age 8.

### Predicting number skills at age 8 using variables at age 7

**Figure 2:**
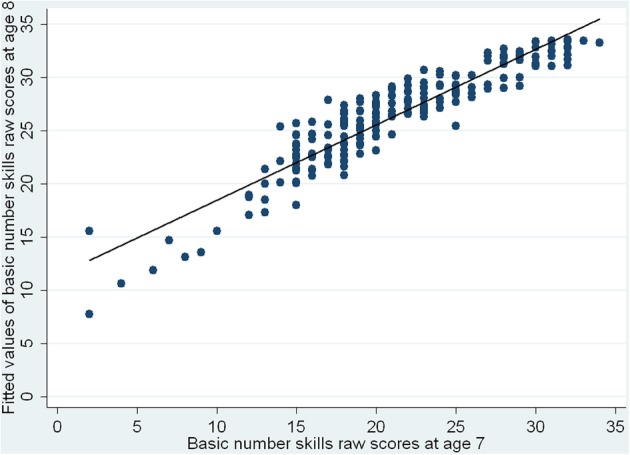
**Fitted values of basic number skills raw scores at age 8 vs. basic number skills raw scores at age 7**.

**Figure 3:**
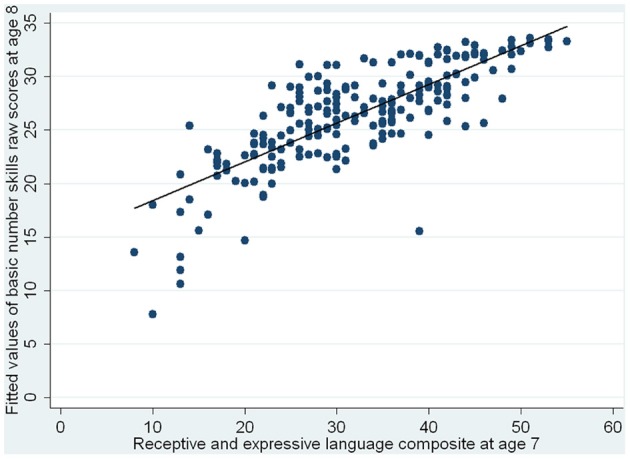
**Fitted values of basic number skills raw scores at age 8 vs. receptive and expressive language composite at age 7**.

Using linear regression analyses and the approach of a conditional change model, we next investigated which variables at age 7 could predict basic number skills at 8. With the reflect square root transformed numbers skills raw scores at 8 as the outcome variable, PIQ raw scores were entered first, followed by basic number skills raw scores at 7, and lastly, the language composite. The results are shown in Table 5. Basic number skills at 7 and language skills at 7 are both significant predictors of number skills at 8, with better abilities in these areas at 7 associating with better basic number skills at 8. The standardized regression coefficients showed that the effect of earlier number skills was greater than that of earlier language skills. Having controlled for PIQ at 7, number skills at 7 accounted for 36% of the variance in basic number skills performance at 8, while language skills explained a further 4.6% of the remaining variance. The results are also illustrated in Figure 2 which shows the fitted values of basic number skills raw scores at age 8 vs. basic number skills raw scores at age 7, and in Figure 3, which shows the fitted values of basic number skills raw scores at age 8 vs. language composite at age 7. As with Figure 1B, we have re-transformed the predicted values back to the original scale of the raw scores for easier interpretation. The figures again reveal that the higher the basic number skills raw scores at age 7 and the better the language abilities at age 7, the better the basic number skills at age 8.

**Table 5:** **Linear regression analysis predicting basic number skills performance at 8 using variables at age 7**.

	***b***	***SE***	**β**	***R*^2^**	**Adjusted *R*^2^**	**Cohen's *f*^2^**
**TRANSFORMED RAW SCORES AT 8^a^**						
*Step 1*				0.161	0.157	0.19
PIQ at 7	−0.09^***^	0.01	−0.40			
*Step 2*				0.522	0.517	1.09
PIQ at 7	−0.03^*^	0.01	−0.13			
Basic number skills raw scores at 7	−0.11^***^	0.01	−0.66			
*Step 3*				0.570	0.564	1.32
PIQ at 7	−0.01	0.01	−0.06			
Basic number skills raw scores at 7	−0.09^***^	0.01	−0.53			
Receptive and expressive language composite at 7	−0.03^***^	0.01	−0.28			

N = 212.

a Reflect square root transformation of raw scores at age 8.

* p <0.05.

*** p < 0.001.

### Predicting the drop in performance in basic number skills between ages 7 and 8

Some improvements in most children's scores could be expected over the course of 1 year. However, some participants failed to show improvement, and some showed a decrease. Investigating why some children are not making progress, or appear to decline, in a particular skills area contributes importantly to our understanding of which factors impede development. Hence, we examined the proportions of children who, from 7 to 8 years, showed no change, or who showed a decrease in scores, or who showed an increase in scores. These proportions were examined with respect to raw scores and standard scores. The results are presented in Table 6. Although 84% had seen an increase in their raw scores, only a third (33%) had seen a rise in their standard scores. In fact, the standard scores of over half of the children (53%) had fallen between the two ages. This again suggested that although the basic number skills for the vast majority of participants had progressed between ages 7 and 8, the rate of progress, on average, was not as great as that of their population peers.

**Table 6:** **Participants showing a change or no change in the basic number skills raw and standard scores between ages 7 and 8**.

	***N***	**%**	**Mean change**	***SD***
**RAW SCORES**				
No change in raw scores	12	5.2	0	—
Decrease in raw scores at 8	24	10.5	−3.5	2.4
Increase in raw scores at 8	193	84.3	6.1	4.0
All	229	100	4.8	4.9
**STANDARD SCORES**				
No change	31	13.5	0	—
Decrease in standard scores at 8	122	53.3	−9.8	6.5
Increase in standard scores at 8	76	33.2	9.0	6.6
All	229	100	−2.2	10.5

Binary logistic regression analyses were carried out to examine which variables could predict a drop in the maths scores between ages 7 and 8. We focus on children who remain static or show a decline on the grounds that these are most likely to yield information on which factors impede development. These are also the children most in need of educational support. The outcome of the regression was coded as “0” (the reference category), representing no change or an increase in raw scores, or “1,” a decrease in raw scores. A variable representing the difference in PIQ raw scores between ages 7 and 8 was first entered into the model, followed by the language composite raw score differences between 7 and 8 years. The results for predicting a drop in performance in basic number abilities are presented in Table 7. The difference in language skills between 7 and 8 years was the only significant predictor in the final model. With an odds ratio of less than 1, results of the final model suggested that the larger the change in language skills between ages 7 and 8, the smaller the odds of a drop in basic number abilities. That is, the greater the improvement in language ability, the lesser the likelihood of a drop in performance in basic number skills from 7 to 8 years: less language improvement, more likelihood of a drop in language skills. With an odds ratio of 0.90, one unit decrease in the difference in language composites was associated with a 11% increase in the odds of having a drop in number skills at age 8. The analogous analysis was performed on standard scores and the pattern of results was essentially unchanged.

**Table 7:** **Binary logistic regression analysis predicting a drop in performance in basic number skills between ages 7 and 8**.

	**Odds ratio**	***SE***	**95% CI**
**STEP 1**			
Difference in PIQ raw scores	0.88^*^	0.05	0.78–0.99
**STEP 2**			
Difference in PIQ raw scores	0.90	0.06	0.80–1.02
Difference in language composites	0.90^**^	0.03	0.84–0.97

N = 208;

* p < 0.05;

** p <0.01.

## Discussion

The purpose of this longitudinal study was to examine the extent to which, in a sample of children with SLI, the severity of their condition predicts progress in number skills development during the early primary school years. Adding to earlier evidence of a disadvantage to children with SLI in this domain (Fazio, 1999; Arvedson, 2002; Cowan et al., 2005; Donlan et al., 2007; Kleemans et al., 2011, 2012), we obtained clear findings that this large sample obtained mean standard scores more than 1 *SD* below the population mean at age 7 and again at age 8. The standard scores of over half of the children (53%) had fallen between the two ages. Overall, then, children with SLI fell well below TD norms at age 7, and the gap worsened by age 8.

Both PIQ and language scores were significant predictors of concurrent number skills at both ages, with better abilities in these areas being associated with better basic number skills. Together, these variables accounted for 36 and 40% of the variance in basic number skills performance at ages 7 and 8, respectively. After controlling for the effect of PIQ, language skills explained an additional 19 and 17% of the variance in the raw scores for ages 7 and 8, respectively. Cowan et al. (2005) found a similar pattern in their analysis of data pooled across TD and SLI participants; the present findings confirm that the relationship holds within SLI. Thus, severity of language impairment is clearly associated with number skills ability.

The next question of interest was whether language ability at age 7 contributed to the prediction of number scores at age 8. We regressed number scores at age 8 on PIQ at age 7, maths score at age 7, and language ability at age 7. This analysis revealed that, after taking account of the other predictors, i.e., PIQ and maths scores, language abilities explained an additional 4.6% of variance in the later number scores. This indicates that, as well as impacting at age 7, severity of language impairment also influences continuing progress in number skills over the following year.

We examined next whether the likelihood of decline in number scores could be predicted from changes in language ability, controlling for changes in PIQ. The change in language skills between 7 and 8 years emerged as the only significant predictor in this analysis. The results were robust and indicated that less improvement in language ability was associated with a greater odds of a drop in performance in basic number skills from 7 to 8 years.

With respect to the contrasting theoretical positions we outlined above, the evidence does not support a strong version of the “separate origins” hypothesis, holding that number and language abilities develop independently. This would predict that progress in number performance should not be affected by the severity of language impairment and the present results support the opposite conclusion. However, the present study, focusing on middle childhood, is moot on the question of early origins and, because all of the number tasks we administered required at least some degree of verbal processing, we do not have data to allow us to compare performance on non-verbal number tasks. Previous research has addressed this issue and found mixed results, with some indicating that children with SLI can perform at around the level of typical peers on non-verbal number tests (Arvedson, 2002; Donlan et al., 2007; Kleemans et al., 2011, 2012), and others suggesting more pervasive deficits (Cowan et al., 2005).

Our results are more readily compatible with theoretical stances holding that the development of number skills is influenced by the child's linguistic abilities. Consistent with this position, our findings that children with SLI are at a disadvantage and show an increased gap from peer norms over the course of a year suggest that language is an impediment to their early engagement with number and a continuing liability as children with SLI face increasingly complex number work in primary education. Further research is clearly warranted to determine if non-verbal number skills progress at a different pace to verbally-mediated number skills in children with SLI, but it is important to bear in mind that, in practice, school work in number and mathematics tends to be immersed in language, both domain-specific and general (Durkin and Shire, 1991; Ginsburg, 2009).

Evidence from children with SLI provides valuable information to help our understanding of both atypical and typical development. The present findings confirm that the child's ability to handle the medium of communication bears on his or her progress in a crucial area of learning, number skills development. The more severe the language impairment, the more difficult the task of mastering number skills. Conversely, we can infer that unimpaired language development in the typical child supports number and mathematical development. This is less readily apparent in typical development because the language ability is assumed and transparent, but recall that Cowan et al. (2005) found that language comprehension was the best predictor of variation on number tasks in a combined sample of TD and SLI participants. Of course, many other endogenous and environmental factors also bear on the complex processes of learning about number, but language is a fundamental component.

It should be acknowledged that the origins of language impairment itself have not been addressed here. It could be argued that both language and number skills development are dependent on similar underlying neural and cognitive processes, such as working memory. This in turn raises questions about the nature of working memory resources (e.g., verbal vs. non-verbal) that might be drawn upon in number tasks (see Nys et al., 2013, for an interesting discussion). While these are broader issues than our present concerns, we reiterate that evidence from children with SLI, who show language difficulties in the context of cognition within the normal range, makes an important contribution to the larger inquiry into the underpinnings of number development.

The present study did not include an age-matched TD sample. Several previous studies have done so and the findings are clear. An advantage of such designs is that the participants are tested contemporaneously, by the same investigators under similar conditions; a possible disadvantage is that the experimenters are not usually blind to participant diagnostic status, raising the possibility of demand characteristics contributing to results. By using a standardized test of number abilities we were able to draw instead on normative data collected from a large representative sample, independently of the present investigators. The fact that similar results are obtained via each comparison strategy supports confidence in the overall pattern of results.

There are obvious but important practical implications that follow from our findings. We found that not only did children with SLI tend to underperform in number skills, relative to standard performance, some remained static over a full school year and a substantial proportion declined. There is the risk that the problems children with SLI face in early mathematics education are compounded over time. The tasks themselves become more advanced and, as others have pointed out (Arvedson, 2002; Cowan et al., 2005), children's difficulties with the verbal demands may be less apparent to educators and therapeutic intervention is likely to be concentrated on linguistic skills. Future research is needed to investigate whether the worsening gap that we have demonstrated here continues to grow over the remainder of primary schooling and beyond, and to assess the efficacy of interventions designed to meet these children's language needs in the particular context of mathematics.

In sum, the present study, with one of the largest SLI samples to be tested on number development in childhood, reinforces earlier findings that children with this disorder are at a disadvantage in this domain. Important novel contributions are that not only do children with SLI fall below peer norms on number tasks but that the gap worsens from age 7 to 8, that language ability contributes substantially to the concurrent prediction of number scores, that language ability at age 7 contributes to the prediction of number scores a year later, and that changes in language ability over the year are associated with whether or not children with SLI manifest a drop in performance in basic number skills at age 8. Language ability underpins progress in number understanding and education.

### Conflict of interest statement

The authors declare that the research was conducted in the absence of any commercial or financial relationships that could be construed as a potential conflict of interest.
